# Students’ perspective on genomics: from sample to sequence using the case study of blueberry

**DOI:** 10.3389/fgene.2013.00245

**Published:** 2013-11-26

**Authors:** Austin B. Mudd, Elizabeth J. White, Michael P. Bolloskis, Nicholas P. Kapur, Koyt W. Everhart, Ying-Chen Lin, Weston W. Bussler, Robert W. Reid, Ryan H. Brown

**Affiliations:** ^1^Plants for Human Health Institute, North Carolina State University, KannapolisNC, USA; ^2^Department of Bioinformatics and Genomics, University of North Carolina at Charlotte, KannapolisNC, USA; ^3^General Mills, Inc., KannapolisNC, USA

**Keywords:** blueberry, sequencing, assembly, mapping, annotation, pathway elucidation, genomics

## Abstract

Advances in genomic sequencing technologies in the past decade have revolutionized the field of genomics, resulting in faster and less expensive sequencing. Holding back the potential for innovation, however, is a widespread lack of understanding of genomics and sequencing by the general public. In an attempt to remedy this problem, this paper presents an introduction to the fields of genomics, bioinformatics, and proteomics using the blueberry genome as a model case study of the plant genomics field. The blueberry (*Vaccinium* sect. *Cyanococcus*) is often cited as a “super food” in the media due to its nutritional benefits and global economic importance. There have been a number of related genomic publications in the past 20 years; however, a completed genome and a full analysis into the health-related pathways are still needed. As exemplified by this blueberry case study, there are opportunities for future genomic research into numerous beneficial plant species. The solid background presented in this paper provides future researchers the foundation to explore these uncharted areas.

## INTRODUCTION

Increased public awareness about the health benefits of blueberries has elevated its worldwide demand in recent years; thus, researchers and breeders seek ways to make blueberry cultivation more efficient and fruitful. An increased understanding of the blueberry genome and pathways, for example, can facilitate selection for climatic adaptation, enabling blueberry growth in new regions as well as greater cultivation in existing growth regions. Through pathway elucidation, blueberry fruit quality can also be improved, providing consumers and breeders with the traits they desire. For consumers, these characteristics include increased antioxidants and dietary fiber, improved taste, cheaper costs, and added vitamin content. For breeders, disease resistance, pest resistance, increased yields, and increased zone hardiness are qualities of interest. For a beginner in this discipline, a clear understanding of mapping, sequencing, genome assembly, and proteomics will provide the necessary framework to explore and contribute intellectually to the growing field of plant genomics. This paper presents an overview of these topics with an emphasis on how they pertain to the study of blueberries.

## OVERVIEW OF BLUEBERRIES

Blueberries provide a perfect case study to explore current research in the fields of genomics, bioinformatics, and proteomics, as it is not one of the highly characterized plant species such as rice (*Oryza sativa*) or *Arabidopsis thaliana*. Humans have gathered and consumed blueberries for thousands of years. Blueberries are native to North America and were a staple in the diet of Native Americans and early settlers. Additionally, blueberries were used for medicinal purposes, relieving fevers, headaches, and persistent coughs (Trehane, 2004). Currently, North America is the largest commercial blueberry producer in the world, producing 600 million pounds of blueberries in 2012. Production in North America has been on the rise, with the total supply increasing by an average of 20% every two years since 2008 (Brazelton, 2013).

### HEALTH BENEFITS

Consumption of blueberries has increased dramatically in the past 15 years due to consumers’ growing knowledge of its health benefits. Blueberries, along with several other berries, contain various types of anthocyanins, which are compounds with anticarcinogenic and therapeutic properties. These compounds have the ability to negate the effects of free radicals in the body, thereby protecting DNA integrity, improving brain function, preventing cancer cell formation, and reducing cardiovascular disease (Zafra-Stone et al., 2007). In fact, a study conducted in rats correlated the consumption of blueberries with protection against neurodegeneration and cognitive impairment. During an 8-week period, rats were injected with kainic acid (KA) and then fed either a diet containing 2% blueberry extract or a control diet. After analyzing a variety of performance variables, researchers concluded that KA-treated rats showed clear signs of impaired learning performance, but the blueberry diet reduced the impairment (Duffy et al., 2008).

### GENETICS AND GROWTH

Blueberries have a varied genetic background. The basic chromosome number (*x*) is 12 (Rowland and Levi, 1994), and seven different ploidy levels have been found in the wild: 2*x*, 3*x*, 4*x*, 5*x*, 6*x*, 8*x*, and 12*x* (Bruederle and Vorsa, 1994). The blueberry genome is estimated to be 500–608 Mb/1C DNA, which is four to five times larger than the 125-Mb *Arabidopsis thaliana* genome and several times larger than other fruiting plant genomes such as the strawberry (240 Mb; Shulaev et al., 2011), grape, and raspberry genomes (Die and Rowland, 2013).

In terms of growth, blueberries require significant organic matter and thrive in acidic, sandy soil, which was originally considered marginal for crop production (Trehane, 2004; Rowland et al., 2012a). There are numerous varieties of blueberry in existence around the world, but four of the major varieties are *Vac-cinium corymbosum* (Northern highbush), *V. darrowii* (Southern highbush), *V. angustifolium* (lowbush), and *V. ashei* (rabbiteye; Trehane, 2004). Different varieties, however, have particular requirements, such as chilling hours. The number of chilling hours is a measure of accumulated hours of temperatures below 7°C in the dormant season (Cesaraccio et al., 2004).

Genetic studies on blueberries have the potential to significantly improve fruit quality and the breeding process. Blueberry breeding will be simplified, for instance, through the use of genetic markers to identify desired seedlings. Rather than waiting for seedlings to mature and then examining their phenotypes, breeders can proactively select and cultivate individuals with desired genotypes, speeding up the screening process. Utilization of genetic markers will make traditional breeding methods more efficient and less random (Hancock et al., 2008).

## SEQUENCING AND ASSEMBLY

Sequencing genomes has the potential to solve large-scale and small-scale biological problems by characterizing key genes involved in various biological processes. These include genes whose mutations lead to disease susceptibility (*NOD2* and Crohn’s disease in humans; Ogura et al., 2001), genes involved in flowering and fruiting (*FT* and *CO* in *Arabidopsis thaliana*; Turck et al., 2008), and genes controlling anti-inflammatory responses (IL6 in humans; Xing et al., 1998). The recent development of easier and cheaper sequencing technologies has led to a rapid increase in the number of sequenced genomes. With regard to plants, *Arabidopsis thaliana* was the first sequenced plant genome in 2000 and has become the model organism for plant genomics due to its small genome size, short life cycle, and low chromosome number. These features are extremely desirable in the sequencing process. When sequencing a new plant species, various elements must be considered including the overall genome size, the presence of duplications and repetitive DNA, and ploidy factors. Crop species, in particular, are inherently difficult to work with due to large, repeated, and duplicated genomes from years of human induced inbreeding (Barthelson et al., 2011).

### EVOLUTION OF SEQUENCING

There has been a proliferation of sequencing processes and technologies over the past decade. Sanger sequencing, the primary sequencing technology for the past 30 years, produces reads of up to 1,000 bases and remains the gold standard for accuracy. Unfortunately, the cost and required time for Sanger sequencing make it prohibitive, particularly given the recent rise of next-generation sequencing. These new technologies include Roche 454 pyrosequencing (2005), Solexa/Illumina (2006), SOLiD (2007), and Helicos single-molecule sequencing (2008). These techniques center on placing millions of DNA fragments on a surface and then sequencing the fragments simultaneously. The fragments, however, are smaller in length, ranging from 25 to 400 base pairs depending on the technique (Pop, 2009). Although these methods are quickly advancing, third-generation sequencing technologies, which have longer-read lengths, shorter run times, and single-molecule resolution, have arisen over the past few years. These third generation technologies include Pacific Biosciences PacBio, Life Technologies Starlight, Oxford Nanopore, and Ion Torrent. Life Technologies Starlight, for example, has a read length of 1,500 base pairs and a run time of 20 minutes (Munroe and Harris, 2010; Egan et al., 2012). More detailed information about the mechanisms, strengths, and limitations of next-generation and third generation technologies can be found in Egan et al., 2012, and a review of plant-focused sequencing is available in Hamilton and Buell, 2012.

### SHOTGUN SEQUENCING PROCESS

Genome sequencing projects in recent years have centered on whole-genome shotgun sequencing. During this process, a genome is broken into small fragments, and a subset that fits a prescribed size range is selected for sequencing from both ends, creating paired reads. Most sequencers utilize paired reads a fixed distance apart to compensate for small read fragment lengths. These sequences are then assembled with the end goal of reconstructing whole chromosomes (Pop et al., 2004b; Pop, 2009). In general, the genome assembly’s quality improves with more reads and depth, which is also known as coverage, or the ratio of total bases sequenced versus genome size (Pop, 2009; Kane et al., 2011). However, an increase in read depth will not solve the current difficulties with sequencing repetitive DNA. Repetitive DNA is a particular problem for computational approaches, as the repeats produce bias, ambiguities, and errors in the assembly process (Treangen and Salzberg, 2011). Various approaches have been suggested and utilized in recent years to avoid this problem, such as parallel sequencing with a similar species (Macas et al., 2007).

### ASSEMBLY PROCESS

Using millions of sequenced short reads, genome assembly combines the reads into contiguous segments (contigs), which are ideally reconstructed into whole chromosomes (Boetzer et al., 2011). Initially, this process compares shotgun sequence reads and overlaps the reads using an indexing technique to identify the sequences that commonly overlap. After indexing, another algorithm aligns similar reads, laying out all of the alignments. To get a final DNA sequence, the layout is condensed down to a consensus (Pop et al., 2004b). Additional algorithms calculate the distance between mate pairs and construct supercontigs, also known as scaffolds, which are multiple contigs joined together (Boetzer et al., 2011). For example, if one end of a mate pair is located in one contig and the opposite end is located in another contig, the distance between the mate pairs ascertains the distance between these two contigs in the genome. Multiple mate pair links are desired in order to corroborate the correlation between two contigs. Since the gap between these contigs lacks sequenced data, “N”s fill the empty space (Pop, 2009).

### *DE NOVO* AND COMPARATIVE ASSEMBLY

Assembly of contigs and scaffolds utilizes two approaches: *de novo* assembly and comparative assembly. In *de novo* assembly, all reads are assembled based on algorithms, and no outside template is used. In comparative assembly, the sequence assembly can be aligned to a reference genome, a BAC (Bacterial Artificial Chromosome) library, or linkage groups. A reference genome utilizes a well-annotated genome that is similar to the species of interest. *Arabidopsis thaliana*, for example, is the primary reference genome for most plant studies. BAC libraries can also be used in comparative assembly. In this, a query genomic sequence of several thousand to over one hundred thousand bases is ligated onto bacterial vector DNA. Bacteria take up the DNA and then replicate on an agar plate. Following this replication, the bacterial DNA with the original query sequence is cut with restriction enzymes, and overlaying of different BACs can determine the restriction enzyme locations for a particular sequence (Shizuya et al., 1992; Shizuya and Kouros-Mehr, 2001). BAC libraries are useful because they only include the actual genome without additional cloning artifacts or alterations from the cloning process (Osoegawa et al., 2001). Finally, mapping of linkage groups can assist in assembly. For instance, mapping of restriction enzyme sites can result in an ordered directory of all restriction fragment length polymorphisms for the genome. The location of these restriction enzyme sites can be matched to the assembled contigs, helping to merge the contigs into scaffolds and eventually chromosomes (Pop, 2009).

### ASSEMBLY PROGRAMS

A variety of programs exist for genome assembly including Arachne (Batzoglou et al., 2002), Bambus (Pop et al., 2004a), Euler (Pevzner et al., 2001), MIRA (Chevreux et al., 1999), Newbler (Margulies et al., 2005), and Velvet (Zerbino and Birney, 2008). These programs can be specialized for contig construction, scaffold building, or both, though most use a greedy approach, which starts with the reliable baseline data and then slowly merges additional data as long as this supplemental information agrees with the current sequence construction. Each assembler has unique strengths and weaknesses, as seen by comparing the MIRA and Newbler assemblers. The Newbler assembler, which is distributed by 454 Life Sciences, fails to account for repeats in an organism’s genome, therefore misassembling the genome but creating fewer and larger scaffolds than the MIRA assembler (Lai et al., 2012). The MIRA assembler flags these repeats but results in a larger number of extremely small scaffolds. In addition, different assemblers work with different read types. For example, the Newbler assembler works best with 454 sequences. When deciding on assembling software, scientists must balance the strengths and limitations of each genome assembly program.

## GENOME MAPPING

Genome mapping is an essential step for assembling highly repetitive genomes. There are two types of mapping: genetic mapping and physical mapping. Genetic mapping approximates the distance between genetic markers by comparing recombination frequencies. Genetic mapping requires cultivation of populations and acquisition of marker data that have been specifically designed for mapping. Physical mapping, on the other hand, anchors a linkage map to physical locations using sequencing, BAC libraries, or restriction enzyme sites (Collard et al., 2005). The end goal of genetic mapping is to create large linkage groups that sufficiently cover the organism’s chromosomes and can be used to assemble the sequence into chromosomes.

### MAPPING POPULATION

To make a genetic map, the distance between loci must be calculated by creating and genotyping the recombination frequencies between loci within a particular population. Accurately estimating recombination frequencies in a population requires a thorough understanding of the species, particularly the reproductive methods. For example, some plants can reproduce by self-pollination, while others require cross-pollination due to self-incompatibility. Using this knowledge, mapping populations create a population of individuals with stable segregation ratios. The genetic markers are then identified on each individual, and the recombination ratios for each marker are plotted in a matrix. The ratios of recombination determine the centiMorgan linkage distances between markers, such that 1 cM equals 1% recombination (Semagn et al., 2006).

### GENETIC MARKERS

Genetic markers represent molecular differences between or within a species and are used to tag particular sequences due to their proximity to genes of interest. Genetic markers distinguish polymorphisms between an individual offspring, assess genetic relationships, and assist in linkage map construction (Collard et al., 2005). As for marker types, RFLPs (restriction fragment length polymorphisms) are differences between homologous DNA sequences that are digested by restriction enzymes. Restriction enzymes cut specific locations on DNA, resulting in different length fragments. RAPDs (random amplification of polymorphic DNA) are DNA segments that are randomly amplified by PCR and total 8–12 nucleotides (Levi and Rowland, 1997). Another type of marker, EST-PCR (expressed sequence tag-polymorphic chain reaction), is more commonly used today. ESTs are short DNA fragments of 200–500 nucleotides and are generated from sequencing one or both ends of an expressed gene, called complementary DNA (cDNA). Since EST markers are transcribed from mRNA, they only contain expressed genes and do not include introns (Dhanaraj et al., 2004). In addition to ESTs, SSRs (simple sequence repeats) have regularly been used in plant genomics. SSRs, also called microsatellites, are short repeated DNA sequences of 2–6 base pairs (Boches et al., 2006).

### BLUEBERRY MAPPING

The first blueberry genetic markers published were RFLPs (Haghighi and Hancock, 1992). This study utilized RFLP segregation in blueberry mitochondrial DNA to distinguish diverse highbush cultivars. Since the 1990s, other genetic markers have been analyzed in blueberry including RAPD (Rowland and Levi, 1994), SSR (Levi and Rowland, 1997), and EST-PCR markers (Dhanaraj et al., 2004). Focusing on EST-PCR markers, the first blueberry EST-PCR study examined cold acclimation genes using fruit, flower bud, leaf, and stem tissue (Dhanaraj et al., 2004). More recently, 110 EST-PCR markers have been mapped into 16 genomic linkage groups (Rowland et al., 2012b). EST markers have also been used in evaluating blueberry species including lowbush and rabbiteye (Rowland et al., 2010). Furthermore, EST library development has assisted with finding blueberry SSR markers (Boches et al., 2005; Bassil, 2012). Continued marker studies will help with identification of blueberry cultivars and management of germplasm in gene banks.

## ANNOTATION

### ANNOTATION PREPARATION

Following assembly of contigs and scaffolds, various test statistics are used to determine if an assembly is ready for annotation. The most widely used statistic is N50, a scoring metric that describes the length of assembled scaffolds (International Human Genome Sequencing Consortium, 2001). The sequences are sorted according to size and then summed from the largest sequences in decreasing order until half of the total size of the sequences has been tallied. The N50 statistic is the size of the smallest contig or scaffold within this set of the largest sequences. Other statistics, such as percent gaps and percent coverage, ensure that there is not significant missing data (Yandell and Ence, 2012). Though N50 is frequently used, it only describes part of the assembly and has been disputed as an ideal metric for describing assemblies (Baker, 2012). Other metrics based on the N50 have also been proposed (Earl et al., 2011; Mäkinen et al., 2012).

### REPEAT MASKING

After a sequence has been declared ready for annotation, repeats must be identified and masked. Repeats are short sequences that occur multiple times throughout a genome. Plant genomes contain a high percentage of repetitive DNA. This repetitive DNA can prevent the computer from gathering evidence and correctly assigning sequence locations, which is especially problematic for sections of coding DNA. Many computer programs can identify and mask repeats, such as RepeatMasker (Tempel, 2012) and RepeatScout (Price et al., 2005), allowing the program to ignore the repeat (Bao and Eddy, 2002). Once the repeats are masked, the process of gene annotation commences.

### ANNOTATION PROCESS

There are two types of gene annotation: *ab initio* and evidence-driven. *Ab initio* gene prediction uses computer-driven mathematical models to identify putative genes and determine their intron-exon structures. These predictions can be advantageous, as they do not require external evidence, saving time and money. Unfortunately, this approach caps the prediction’s accuracy at 70%. Evidence-driven gene annotation, on the other hand, uses data obtained from further analysis. This additional data, which can include gene expression using the transcriptome or ESTs, protein isolation, or experimental evidence based on cloning and characterization, results in a more accurate approach to gene prediction. This accuracy, however, comes at a price, being more costly and time intensive (Yandell and Ence, 2012). For a less expensive and time-consuming method, automated tools such as BLAST (Basic Local Alignment Search Tool) can query an input of known genes against the unknown genome and locate similarities between the two samples (Altschul et al., 1990). These results must be individually examined and interpreted in light of additional *ab initio* and evidence-based predictions to obtain the final genome annotation.

### PATHWAY ELUCIDATION

Pathway elucidation, which involves building gene expression pathways and discovering the identities of biochemical compounds, can provide insights into the biosynthesis of under-investigated natural products. Specifically, many studies analyze comparative transcriptomics and gene expression to characterize biosynthesis pathways. Following the introduction of stress to an organism, random mutations arise, potentially altering the natural processes and genetic functions. These conditional stress factors often test a plant’s ability to tolerate adverse conditions. By examining the alterations in the organisms, scientists can take a “top-down” or “bottom-up” genetics approach by studying phenotype to DNA or DNA to phenotype respectively (Fiehn, 2001). This strategy and others, such as target analysis, profiling, fingerprinting, and metabolomics of ESTs and RNA-seq data, enable deeper insight into an organism’s biomolecular pathways (Hirai and Saito, 2004).

### BLUEBERRY PATHWAYS

For blueberries, the anthocyanin and flavonoid biosynthesis pathways are a primary focus of research due to their health and anticarcinogenic properties. A recent study produced 1,000 transcripts and 800 transcription factors relating to antioxidant biosynthesis and identified 90 expressed genes involved in anthocyanin metabolism regulation (Li et al., 2012). A similar study examined the flavonoid biosynthesis pathway (Zifkin et al., 2012). These studies are a first step toward characterizing important metabolic pathways, but they centered on *Arabidopsis thaliana*, which is a distant relative to the blueberry, for their annotations. This distance results in a lack of conservation that could prevent full elucidation of the metabolic pathways. Future investigations should place focus on a closer relative to the blueberry, such as the grape (*Vitis vinifera*), and strive for full elucidation of the metabolic network interactions.

## BLUEBERRY GENOME

Dr. Allan Brown of North Carolina State University is currently working on a draft genome of diploid *V. corymbosum*. Dr. Brown’s *de novo* approach utilizes both Roche 454 and Illumina GAIIx libraries. The working assembly consists of approximately 500 million base pairs, which encode 25,000 genes. Though the genome is not yet published, these recent developments will be valuable for understanding blueberry cultivation, pathways, and nutritional value (Die and Rowland, 2013).

## CONCLUSION

As the disciplines of genomics, bioinformatics, and proteomics evolve, clear introductions can quickly become convoluted. For novices interested in these fields, a baseline understanding of key concepts is essential. This background will enable these individuals to explore the currently relevant subjects, such as the sequencing of organisms or the elucidation of pathways. In fact, much is still unknown about many currently published genomes. Even the most complete and understood genome, the human genome, is only 10% characterized (Maher, 2012). Additionally, there are many organisms that have not yet been investigated. Examination of new organisms will yield breakthroughs and game-changing discoveries. Of the many organisms with a paucity of research, the blueberry is especially interesting, as scientists and consumers are conscious of its health benefits. Further exploration into the blueberry genome will allow researchers to relate genotype to phenotype, thus providing scientists and farmers with the necessary knowledge to produce blueberries that are more nutritious and desirable.

## Conflict of Interest Statement

The authors declare that the research was conducted in the absence of any commercial or financial relationships that could be construed as a potential conflict of interest.

## AUTHOR CONTRIBUTIONS

Austin B. Mudd, Elizabeth J. White, Michael P. Bolloskis, Nicholas P. Kapur, Koyt W. Everhart, and Ying-Chen Lin contributed to the research, writing, and revision of this paper. Ryan H. Brown oversaw the revision process as the paper’s corresponding author. Weston W. Bussler and Robert W. Reid provided critical reviews in the revision process and helped rewrite subsections of the paper. Austin B. Mudd spurred the initial idea behind the paper and led the overall process.
